# Deformation Analysis of a Composite Bridge during Proof Loading Using Point Cloud Processing

**DOI:** 10.3390/s18124332

**Published:** 2018-12-07

**Authors:** Patryk Ziolkowski, Jakub Szulwic, Mikolaj Miskiewicz

**Affiliations:** Faculty of Civil and Environmental Engineering, Gdansk University of Technology, Gabriela Narutowicza 11/12, 80-233 Gdansk, Poland; mmisk@pg.edu.pl

**Keywords:** civil engineering, geomatics engineering, point cloud processing, sensor fusion, structural diagnostics

## Abstract

Remote sensing in structural diagnostics has recently been gaining attention. These techniques allow the creation of three-dimensional projections of the measured objects, and are relatively easy to use. One of the most popular branches of remote sensing is terrestrial laser scanning. Laser scanners are fast and efficient, gathering up to one million points per second. However, the weakness of terrestrial laser scanning is the troublesome processing of point clouds. Currently, many studies deal with the subject of point cloud processing in various areas, but it seems that there are not many clear procedures that we can use in practice, which indicates that point cloud processing is one of the biggest challenges of this issue. To tackle that challenge we propose a general framework for studying the structural deformations of bridges. We performed an advanced object shape analysis of a composite foot-bridge, which is subject to spatial deformations during the proof loading process. The added value of this work is the comprehensive procedure for bridge evaluation, and adaptation of the spheres translation method procedure for use in bridge engineering. The aforementioned method is accurate for the study of structural element deformation under monotonic load. The study also includes a comparative analysis between results from the spheres translation method, a total station, and a deflectometer. The results are characterized by a high degree of convergence and reveal the highly complex state of deformation more clearly than can be concluded from other measurement methods, proving that laser scanning is a good method for examining bridge structures with several competitive advantages over mainstream measurement methods.

## 1. Introduction

In recent years, significant changes in structural diagnostics have been observed, mostly thanks to the development of remote sensing techniques. Moreover, along with the growing influence of computer science, the processing of remote sensing data, such as point cloud data, has become of greater importance. Especially vivid is the progress achieved in laser scanning technology. High-Performance Terrestrial Laser Scanners can gather one million points per second and have a range of more than one kilometer. New challenges demand more sophisticated methods of point cloud processing, designed to evaluate structures and structural deformations. In this paper, we present a general framework for studying the structural deformations of bridges, especially those that deform in an irregular way, such as composite bridges. We describe in detail the advanced shape analysis achieved with the use of a precise optical device, the terrestrial laser scanner (TLS), along with point cloud data processing. The procedure we present combines rough change estimation, virtual visual inspection, and an extensive spheres translation method (STM) analysis, which allows us to obtain a quick change estimation and a detailed picture of the deformation under different types of load. We evaluated a composite pedestrian foot-bridge during proof loading, subjected to various load cases. The load cases of the proof loading were static, so there was no bridge resonance to consider. The study was carried out as a part of broader research project conducted by the Faculty of Civil and Environmental Engineering at the Gdansk University of Technology. The algorithms and procedures described in the following paper are an extension of the methods designed by the authors in previous studies. We compare the state-of-the-art point cloud processing approach with well-known measurement methods, such as a total station measurement, or inductive sensors measurement. A complex form of the span and its unobvious deformation state allow contributions from the advantages of remote sensing techniques. The greatest gain of TLS usage and its competitive advantage over other measurement methods, such as a total station, is the complexity of the obtained data—TLS creates a three-dimensional projection of the scanned object in time. Using a total station, it is possible to measure one particular point at a time, which extends the measurement time and is more time-consuming. The rationale behind performing the analysis on a composite bridge is that composite bridges deform irregularly in three axes, so using TLS in this particular application is valuable. What is more, the presented STM approach has previously only been used in laboratory conditions.

The topic of bridge evaluation using TLS appears in the international literature. Riveiro et al. [1] used TLS scans and orthophotographs to evaluate masonry arch bridges. Additionally, Riveiro et al. [2] proposed the use of a hybrid method of TLS, photogrammetry, and total station measurement for the structural inspection of the bridge. Xu et al. [3] and Yang et al. [4,5] present an adoption of TLS technology in the deformation analysis of a composite arch structure under monotonic load. They tested a masonry arch on reinforced concrete supports and used a Z+F laser scanner (Zoller + Fröhlich GmbH, Wangen, Germany). The approximated surface model of supposedly the bottom of the lower vault was the focal point of consideration. They calculated the surface difference by the comparison of two epoch surfaces, but they did not mention the exact point cloud processing method that allows calculation of their results. There are a few works which present a field case study of bridge evaluation; for example, TLS. Kitratporn et al. presented an evaluation of a suspension bridge in Myanmar [6]. To measure the steel tower inclination by extracting the planer surface using RANSAC (see Appendix A, Table A1)algorithm [7] they took the average vertical coordinate value of each point on the extracted planer with 1.5 m increments. Zogg et al. used terrestrial laser scanning for deformation monitoring on the Felsenau Viaduct in Switzerland during load tests [7]. They obtained the difference between point clouds by calculating residuals as the shortest distances from the scan points to the reference surface, which was generated by triangulation.

## 2. Materials and Methods

### 2.1. Composite Bridge Description and Experiment Set-Up

**Figure 1:**
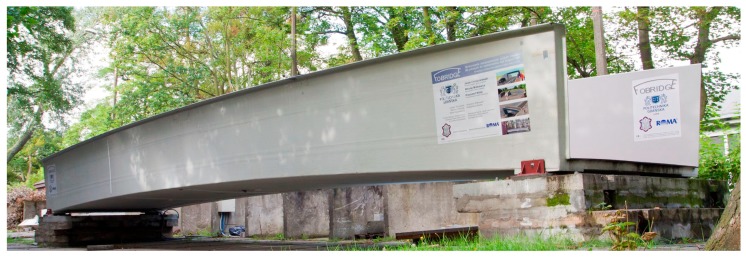
Composite bridge.

**Figure 2:**
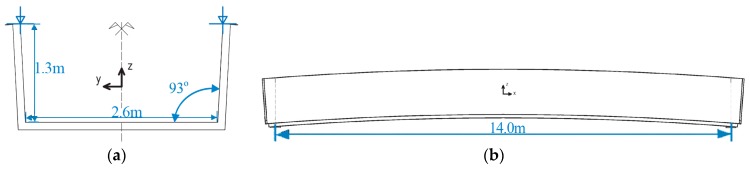
Composite bridge cross-section (**a**) and side view (**b**) with dimensions.

Composites compete with standard materials like concrete, steel, or wood. Composites are primarily much lighter than conventional materials and do not erode, which is crucial for constructions exposed to an aggressive environment. In the considered bridge, the spans have a sandwich-type support structure. The core is foam and coatings built from laminated fiber-reinforced polymer in the form of sandwich panels. The sandwich panel skins and lips are from the flame-retardant vinyl-ester resin as a matrix, and E-glass fabrics as its reinforcement. The polyethylene terephthalate foam core of the sandwich panel has a thickness of 100 mm and a density of 100 kg/m^3^. Due to significant local actions, which cannot be sustained by foam, the core in the support area consists of fiber reinforced polymer. The bridge in the longitudinal and transverse directions has chopped strand ribs inside the core. The total mass of the footbridge superstructure is 3200 kg. The bridge has a low-elevation pseudo-arch, simply supported by the span of a U-shape channel section with auxiliary lips. The bridge was designed to meet specific parameters, such as the weight, bearing capacity, and conditions needed to achieve specific bearing capacity, comfort of use, attractive architectural design, durability, non-flammability, weather resistance, UV radiation resistance, chemical and electrical insulation, ease of assembly and disassembly, and easy repair and maintenance. The composite bridge span is one of the unique constructions made entirely as one piece. This method involves vacuum resin impregnation and thanks to the infusion process, color, texture, and the decorative element can be included, which does not exclude casual surface painting. The bridge was devised and assembled within the “FOBRIDGE” project (Gdansk University of Technology: Project Leader, Warsaw Military University of Technology. Roma Private Limited Company: footbridge manufacturer). More information about the project can be found in the references [8,9]. The image of the bridge, along with the schematic cross-section and side view, are shown in Figure 1 and Figure 2a,b, respectively.

**Figure 3:**
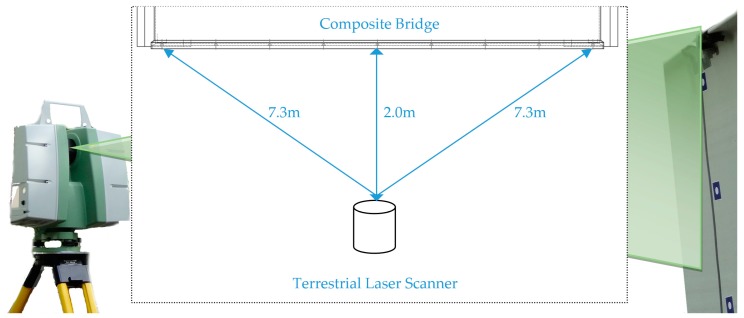
Experimental set-up scheme.

**Figure 4:**
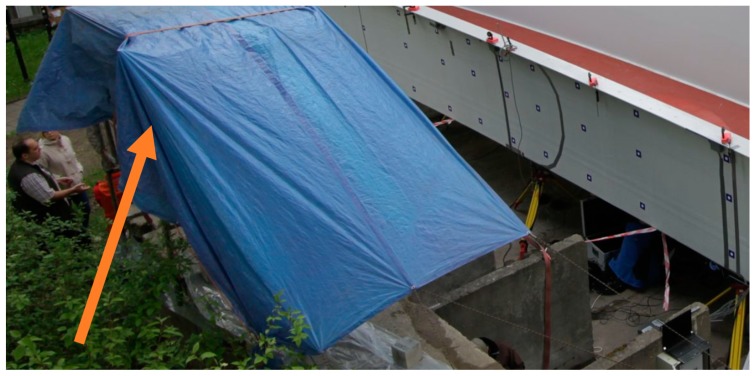
Fixed scanner position: the arrow points out the location of the scanner.

The experimental set-up was in the middle of the span, two meters from the center of the bridge diaphragm. The scheme of the measurement station is shown in Figure 3. The position of the scanner is fixed, as shown in Figure 4.

### 2.2. Measurements and Point Cloud Processing

This chapter describes measurements, point cloud processing, mesh modeling methods, and change detection methods essential for qualitative deformation assessment. We performed scans at point zero, before proof loading and during the proof loading of the composite bridge. The proof loading consists of loading the deck with given load combinations and observing the deformation of the object. We make a scan with every change of the load combination. Due to difficult measuring conditions, especially the large research group that were simultaneously conducting other tests, partially covering the object while the device was sending a laser beam, we performed the TLS measurement multiple times for some load increments. We used a ScanStation C10 scanner, manufactured by Leica Geosystems AG (Heerbrugg, Switzerland).

#### 2.2.1. Pre-Processing of the Point Cloud

**Figure 5:**
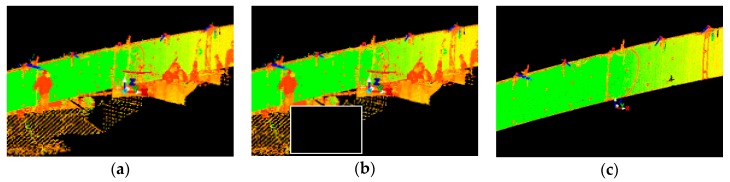
The images show a view of the point cloud in the following phases: (**a**) before pre-processing; (**b**) fenced area for deletion; and (**c**) after pre-processing.

We must process obtained point cloud samples in a certain way before the analysis. Most of the studies which focus on the processing of the point cloud mention three general steps: data sampling, noise reduction, and shadow filling. Data sampling helps to reduce the input redundancy, and its roots can be traced to clustering by Schreiber [10] and Thinning algorithms by Floater et al. [11]. Hou et al. [12] presented an entirely new approach where sampling is carried out by a virtual adaptive process. One of the excellent works on automation of noise reduction is by Fua et al. [13], in which the authors use it for the unstructured point cloud. There are many approaches to noise reduction, such as in Rusu et al.’s work [14], where the authors proposed using a sophisticated algorithm which consists of filtering the point cloud, removing outliers, and returning the linear indices to the points that are either inliers or outliers. This method eliminates noise and resamples the data without deleting the essential details. The shadow filling can be handled by performing additional scans, but there is a method that uses volumetric diffusion, developed by Davis et al. [15]. Raw point cloud data obtained directly after the measurements has to be processed with cleaning tools, and this so-called cleaning involves deleting redundant areas in the point cloud. Excessive regions of the point cloud contain data which do not directly refer to the considered scanned object, such as people, terrain, and trees, as in Figure 5a. We used the manual fencing procedure. The first step is to select an excessive part of the point cloud using a rectangular field, and then to remove everything in this field. The method is usually repeated a few times, as in Figure 5b. The result of the cleaning process is a point cloud representing only the considered object, as in Figure 5c.

**Figure 6:**
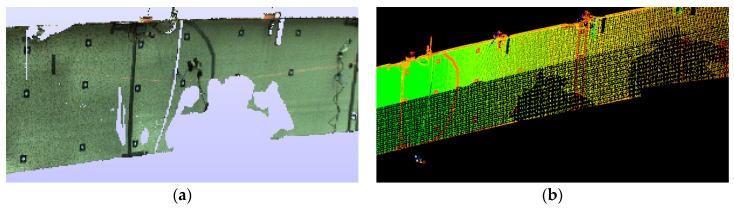
Images refer to point cloud structural processing: (**a**) so-called shadow as a breach in the point cloud; and (**b**) filling the “shadow” with points acquired in additional scans.

Due to various additional measurements that were carried out during proof loading, the lateral surface of the bridge was often obscured by the people crossing the view line between the bridge span and the scanner device. Obstacles between the bridge and a scanner caused the formation of rifts in the point cloud, so-called shadows, as shown in Figure 6a. These may result in an unstable distribution of points in the point cloud. We predicted the occurrence of such a situation, which is why we made several scans for each load case change. Points acquired in additional scans were used to fill the rift, as shown in Figure 6b.

We merged the additional scans by allocating them in the same model space, as a given load case original scan. It is worth emphasizing once again that the position of the scanner was fixed during additional scans, as well as during the entire proof loading process. Effects of the unevenly distributed point cloud may be visible in the form of local congestion and rarefaction of points in the cloud. Point cloud optimization may help to improve redistribution of points in the cloud, but due to the precise nature of the analysis, the authors decided not to interfere with the structure of the points in order to reflect, as closely as possible, the actual state of the deformation in time.

#### 2.2.2. Post-Processing of the Point Cloud

Mesh Generation

**Figure 7:**
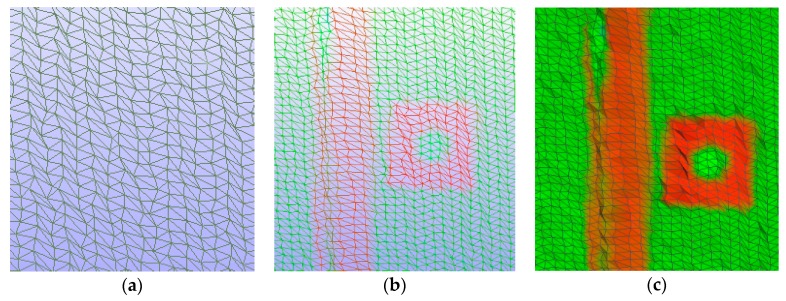
Images refer to point cloud structural processing: (**a**) TIN situated on the point cloud, (**b**,**c**) and the intensity map imposed on a TIN (see Appendix A, Table A1) grid.

There are many methods for mesh modeling of point clouds, and this issue is constantly being developed. The first algorithms which referred to mesh modeling of the point cloud were created by Boissonnat et al. in the mid-1980s [16,17], but were practically not developed further by the scientific community until the beginning of the 1990s, when Hoppe et al. published extensive work about surface reconstruction of the unprocessed point cloud [18]. Intensive work on this issue at the end of the nineties and later resulted in the emergence of a large number of new algorithms, but also a division into two main trends. The first trend focused on the methods where the zero-set of a scalar 3D function estimated the mesh surface [19,20,21,22], and another group used the Delaunay complex to rough mesh surface by its subcomplex [23,24,25,26,27,28,29,30,31]. Modern meshing algorithms mostly perform construction of the Delaunay complex in an incremental manner, and to improve data locality optimize the insertion order by spatial sorting techniques [32,33,34]. A good example of the further development of these algorithms is the use of three point-insertion sequences for incremental Delaunay tessellations performed by Gonzaga et al. [34]. After the pre-processing stage, each point cloud was exported in PTX format with its intensity map for further processing in “MeshLAB” and “CloudCompare”. The software uses an algorithm which connects every spatial point with its nearest surrounding points and builds a triangle grid to create a mesh model for every state in time, as shown in Figure 7a. Several mesh models were prepared, including models covered with intensity maps, as in Figure 7b,c.

How to Detect Deformations in the Point Cloud

It is necessary to compare the reference scan and the scan from a given load case to determine the deformation state of the bridge. There are two groups of point cloud processing methods for change analysis: region-based and point-based. One of the first approaches to TLS data change detection was proposed by Girardeau-Montaut et al. [35] and focused on the direct comparison of point clouds by an average distance, best fitting plane orientation, and the maximum length among the points in one set to the closest point in another set—so-called the Pompeiu–Hausdorff distance. Girardeau-Montaut et al. showed that among these three parameters, the third one gives the best validation. Lindenbergh and Pfeifer [36] presented a solution to detect deformation using an analysis based on the point-to-plane approach, in which points and fitted planes are compared between consecutive epochs. Comparison with the use of range segmentation was presented by Zeibak and Filin [37], who tried to overcome two main issues of TLS data: occlusion and spatial sampling. The method based on point-to-point measurement of the Pompeiu–Hausdorff distance was proposed by Kang et al. [38], and the authors pointed out that point-to-point is sensitive to local point density, tending to make the point-to-plane approach more reliable. Zhang et al. [39] detected a spatial change using an anisotropic-weighted ICP (A-ICP) (see Appendix A, Table A1) algorithm, and also presented how to model the random error. The authors were able to estimate the synthetic surface ruptures. Ziolkowski et al. [40,41] proposed to study the change of the scanned object in time by tracking the position of physical characteristic objects projections. The authors used this method to study the deformations of the concrete element under monotonous loading.

## 3. Results: Analysis of Shape Deformation

### 3.1. Deformation of Bridge Diaphragm during Proof Loading

**Figure 8:**
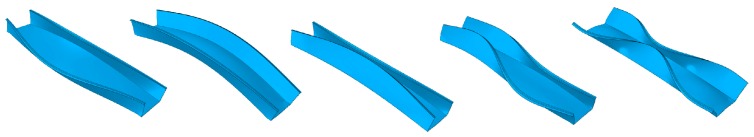
General modal states of the composite bridge [9].

Deformation of bridge diaphragm is particularly crucial for the overall bearing capacity of the bridge. The lateral surface of the bridge diaphragm deformed irregularly, making it difficult to obtain a complete image of deformation with standard measurement methods. The general modal states are the best illustration of various deformation states [9], shown in Figure 8.

**Figure 9:**
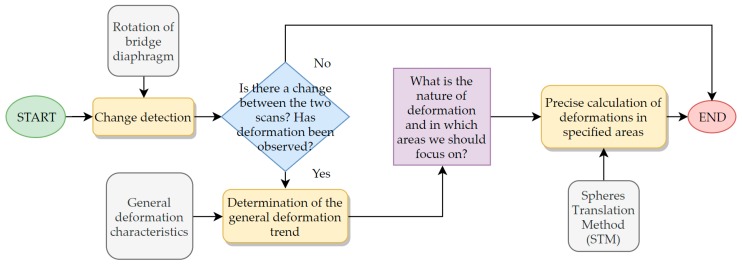
Block diagram of the proposed framework.

We propose a general framework for deformation analysis: the block diagram presented in Figure 9. The solution consists of three stages: change detection, determination of general deformation trend, and precise determination of deformations in specified areas.

### 3.2. Change Detection

#### 3.2.1. Rotation of the Bridge Diaphragm

To determine if a deformation exists, we check whether a change has occurred between the scan before and after applying the load. In the case of the considered composite foot-bridge, deformation of the lateral surface of the diaphragm is particularly essential. The diaphragm rotation between two states in time is a simple valve, which can be used to estimate if the change occurred. However, such a considerable simplification loses details of local deformations, so the tool should be used with caution. The method consists of the creation of two mesh models generated by special kinds of algorithms, called FM (Fast Marching) and KD-Tree. Mesh models, as previously noted, are projections of the bridge diaphragm for two states in time. In the considered case, we used the FM algorithm because it needs fewer parameters and is easier to implement. However, KD-Tree is an excellent alternative to FM.

#### 3.2.2. FM (Fast Marching) and KD-Tree Algorithms in the Building of the Mesh Model

The FM algorithm divides the initial point cloud into smaller patches and regroups them with systematic subdivision, which is not recursive. Afterward, most of the pieces will be the same size, but have different surface curvature due to the set resolution. Next is the fusion process, which is based on FM front propagation. The algorithm assumes that two input parameters, such as the grid resolution, expressed as the subdivision level of the cloud octree and accuracy level, is achieved by re-computation of the facet retro-projection error. We can use an octree for a faster initialization. Another algorithm which is also satisfactory for mesh generation is the KD-Tree algorithm. The algorithm recursively divides the point cloud into small patches in a planar shape, which regroups to larger facets. The method needs several input values, which are as follows: maximum angle between proximity patches, maximum relative distance, maximum angle, current facet center, and the maximum distance between patches, which should be merged. The critical differences between the KD-Tree and FM algorithm are as follows: The subdivision is systematic in FM and not recursive as in KD-Tree. The FM fusion process is based on front propagation. KD-Tree represents a disjointed partition. We decided to use FM because it needs fewer parameters and is easier to implement.

#### 3.2.3. Actual Rotation of the Bridge Diaphragm

**Figure 10:**
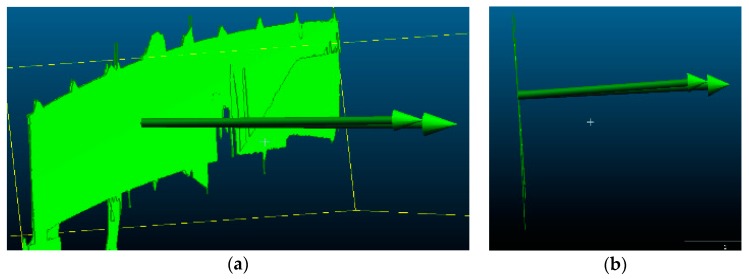
Facets the composite bridge diaphragm generated by the FM algorithm: (**a**) axonometry; and (**b**) side view.

We calculate the actual rotation of bridge diaphragm with use of two mesh projections of the bridge in time, before and after the applied load. Meshes for both states in time were generated by the FM algorithm, with the parameters needed to create estimated facet (shown in Figure 10a,b). We presented the rotation by the surface centers and normal vectors, as shown in Table 1.

This rough simplification allows estimation of whether the change in the element position occurred. The conclusions were that due to the rotation, the upper part of the lateral surface moved in the perpendicular direction, continuously otherwise a bottom portion of the plate. The rotation is clearly visible in Figure 10b.

### 3.3. Determination of the General Deformation Trend

When we found out that the change occurred, we were able to assess the general deformation trends. This should tell us which part of the bridge is the most deformed and on which area we should focus for precise calculation. In this subsection, the authors show what the visual assessment of bridge diaphragm mesh models looks like at two points in time. Selected projections of the bridge at two points in time were placed in one coordinate system and superimposed on each other. The procedure requires a fixed coordination system. By visually analyzing the first image of Table 2-1, it can be seen that these two scans do not cover in a consistent, systematic manner. The support and bottom area of the composite bridge span have a uniformly penetrating grid structure, as seen in Table 2-2. In Table 2-3 patches of different colors permeate with each other, which indicates that no deformation has occurred. However, the middle and upper part of the span do not have the same appearance. The two meshes do not overlap, and the color of only one grid is visible, as shown in Table 2-4. In Table 2-5, the authors managed to capture the curve that the object leans towards from the plane of the bridge diaphragm’s lateral surface with increased deformation, as well as the rotation of the diaphragm lip in Table 2-7. More detailed observations of the bridge diaphragm deformation are in Table 2. This part of the analysis should yield an answer to the deformation trends and on what areas we should focus during the exact calculation of the deformation volume in the next part of the study.

### 3.4. Spheres Translation Method (STM)

#### 3.4.1. Application of the Spheres Translation Method

**Figure 11:**
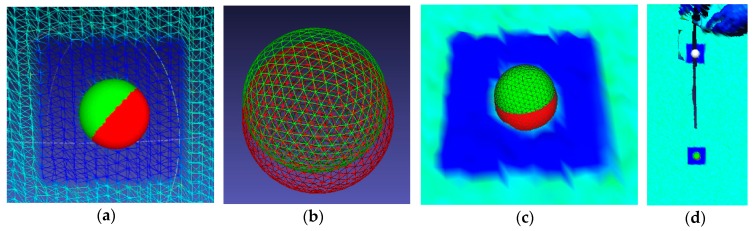
Spheres translation method: (**a**) mesh grid of the bridge diaphragm surface with displaced spheres; (**b**) mesh grid; (**c**) bridge diaphragm surface with displaced objects; and (**d**) flat signals on the bridge diaphragm surface.

To accurately measure the deformations of the lateral surface of the bridge diaphragm, we adapted and used the spheres translation method. The spheres translation method (STM) is one of the point cloud processing procedures, alongside such methods as the point-to-point, point-to-surface, or surface-to-surface methods. The spheres translation method (STM) procedure consists of several steps. The first step is the placement of special tags (e.g., round plates) on the object. Then, during the measurements, we scan the tags along with the entire structure. In post-processing we fit the spheres into the points that represent these tags in the point cloud. Then, we track changes in their position over time. Positional deviations of the spheres indicate the direction of deformation. In other words, it comes down to the selection of characteristic points, which represent the tags on the surface of the object, partially shown in Figure 11d. Transformation of these points to the virtual mesh sphere are tracked in time. This method has several boundary conditions, the most important of which is a fixed coordination system, common to subsequent measurements. The uniform coordinate system was obtained by performing all measurements from a fixed scanner position. We made a “zero” scan before applying the load, which was the reference scan. We identified differences in the position of the spheres as the displacement vector in a given direction. The displacement of the sphere is visible in Figure 11a–c.

We present an example of the location change of the sphere SD_2 over time to illustrate the procedure. We distinguished the spheres marked as SD2_S1 and SD2_S2 by the colors green and red, respectively. The coordinates of both objects are in Table 3. We determined coordinates in respect to the reference scan. The displacement is about 1 mm.

We performed the proof loading (U1) in several steps, presented in Table 4. The overall weight of the slabs in the U1 test was equal to 14,400 kg. Analysis using the spheres translation method (STM) was performed for various loads conducted in the following order: 1 + 2; 1 + 2 + 3; 1 + 2 + 3 + 4; 2 + 3 + 4; 3 + 4; 4. The bridge span was loaded and unloaded alternately.

#### 3.4.2. Comparative Analysis

**Figure 12:**
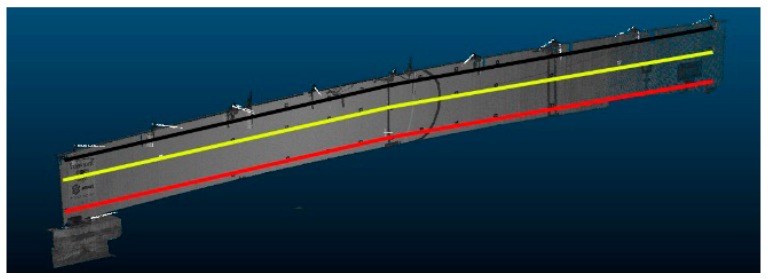
Three analyzed sections on the lateral surface of the bridge diaphragm.

We prepared a comparative study of TLS results with those of the deflectometer and total station. Deflectometer inductive sensors were set at three points below the surface of the bridge span, distant from each other by 3.50 m, and were used to determine vertical displacements. We measured horizontal and vertical movements with the Leica Nova MS50 surveying station. We carried out the spheres translation method (STM) deformation measurements based on TLS data. To statistically describe the deformations of the composite bridge diaphragm lateral surface and enable comparative analysis with other methods, the authors decided to isolate three cross-sections for each analyzed load case, as shown in Figure 12.

**Figure 13:**
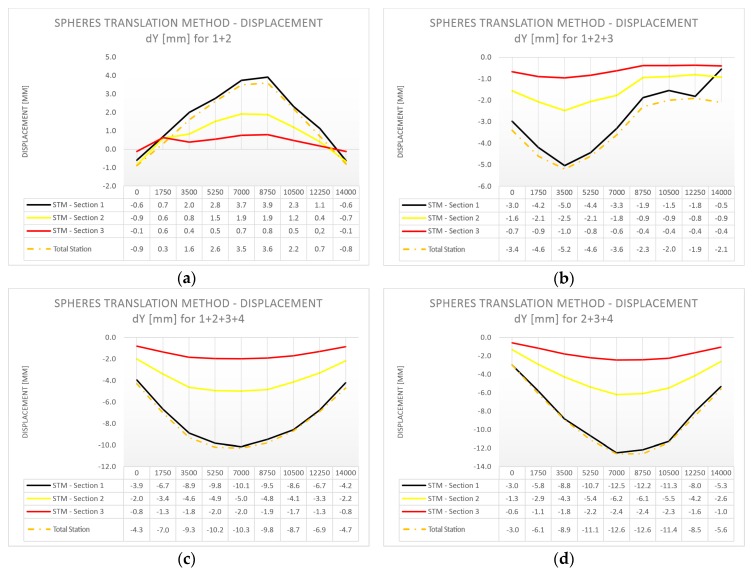

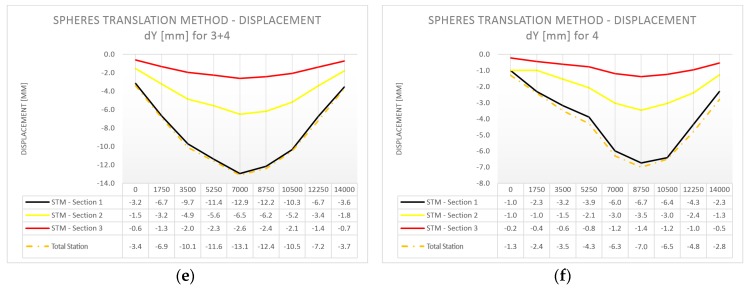
Deformation of the bridge diaphragm in the perpendicular direction during proof loading: STM in three sections and total station (mm); (**a**) Load U1: 1 + 2; (**b**) Load U1: 1 + 2 + 3; (**c**) Load U1: 1 + 2 + 3 + 4; (**d**) Load U1: 2 + 3 + 4; (**e**) Load U1: 3 + 4; and (**f**) Load U1: 4.

**Figure 14:**
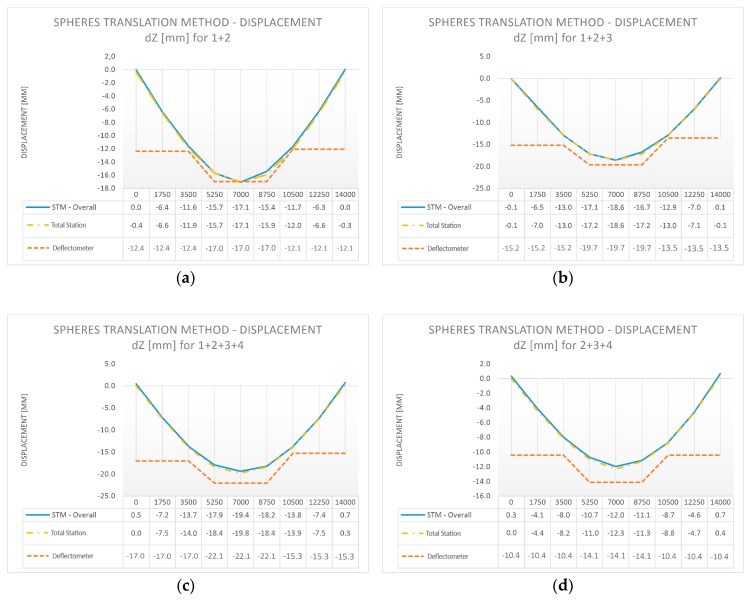

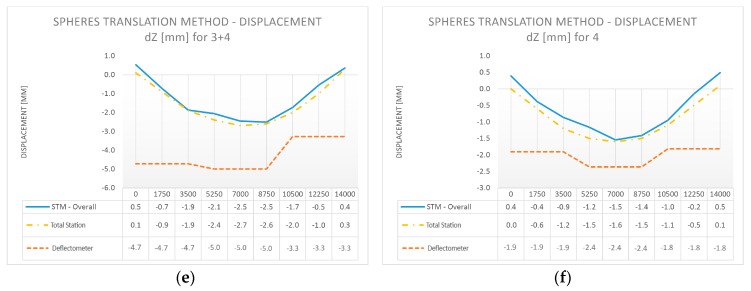
Displacement of the bridge diaphragm in the vertical direction during proof loading: STM in three sections + total station + deflectometer [mm]; (**a**) Load U1: 1 + 2; (**b**) Load U1: 1 + 2 + 3; (**c**) Load U1: 1 + 2 + 3 + 4; (**d**) Load U1: 2 + 3 + 4; (**e**) Load U1: 3 + 4; and (**f**) Load U1: 4.

The profile position was based on the approximate position of spheres located in the closest vicinity of the cross-section. We calculated the displacements of the spheres by applying results from individual load cases to a reference sphere’s position from the case before the load was applied. We present the deformations of the composite bridge diaphragm lateral surface in the perpendicular direction for different load cases in Figure 13a–f (STM in three sections compared with total station measurements), and the vertical displacements in Figure 14a–f (STM in three sections, total station measurements, and deflectometer). We show the data for individual areas of the composite bridge diaphragm lateral surface, which is divided along the length of the bridge into equal sections with lengths of 1.75 m. We decided to adopt the length of 1.75 m because this value corresponds to the placement of markers used for total station measurements.

#### 3.4.3. Observations from the Comparative Analysis

By analyzing the research material presented in Figure 13 and Figure 14, it can be concluded that there is a high convergence of results between the results of displacements obtained by TLS and the measurements from the total station and the deflectometer. A great advantage of TLS over the other measurement methods is a more comprehensive form of results. The use of TLS and STM allows control of the deformations on the whole lateral surface of the bridge diaphragm.

## 4. Summary and Conclusions

This paper presents a general framework for the deformation study of bridges, with a clear indication of the bridges that are subject to very irregular deformation, such as composite bridges. We propose a solution which combines rough change estimation, virtual visual inspection, and STM analysis, giving the advantages of both quickly change estimation and precise deformation measurement. We describe the test set-up configuration, the procedure of pre-processing and post-processing of the point cloud data, and the extensive literature review for point cloud processing, mesh modeling, and change detection. We gathered point cloud data during the proof loading process from a fixed scanner position by a Leica ScanStation C10 terrestrial laser scanner. We performed the first scan before and after the load of the bridge for the various load cases during proof loading. Our algorithm has three steps: The first step is to check if there has been a change in the considered object between the two points in time. We did a quick, rough assessment of whether the change occurred in the object by comparing two facets generated with the FM algorithm. Rotation of facets for different points in time indicates the occurrence of a deformation. Once we know that a deformation exists, we can perform a virtual visual inspection of the bridge by superimposing two mesh models in one model space to see the nature of the distortion. Checking the kind of deformation gives knowledge in which areas it is worth focusing on during accurate measurements, such as STM analysis, as these are time-consuming. We presented how to efficiently perform a virtual visual inspection of the bridge for two points in time. The third step is taking accurate measurements using point cloud processing. We adapted the STM to perform a detailed analysis of the deformations and adjusted the method for field use. We modified the STM by analyzing the object in three sections, which helped to cover most of the bridge diaphragm surface. The method was designed for concrete element deformation under monotonous load in our previous studies [40]. We compared the results from the STM with the results obtained using a total station and the deflectometer, and found they were similar. The advantages of the method proposed by us are a much broader insight into the deformation state of the object for different load cases in comparison with the total station and the deflectometer, which is especially significant in the examination of composite bridge diaphragms as they deform irregularly in a direction perpendicular to the diaphragm lateral surface. Additionally, TLS measurements are much faster than those taken with Total Station and the deflectometer. The downsides of this solution are the sensitivity to changes in the position of the scanner, weather conditions, point cloud density fluctuations, rifts in the point cloud, and improper scanning, and the need for troublesome data processing. The issue of complex shape analysis for the composite structures presented in this paper is significant, and we would like to develop it further. Further work will include the designation of procedures for large-scale bridges, as well as the improvement of existing methods.

## Figures and Tables

**Table 1:** Mesh plane data from both points in time, after facet generation (m).

Epoch	X Coordinate	Y Coordinate	Z Coordinate
Surface center			
1	–2.112770	1.403070	–0.054039
2	–2.132400	1.373320	–0.056683
Normal vector			
1	0.835015	–0.544994	–0.075702
2	0.835581	–0.546797	–0.053072

**Table 2:** Visual analysis of the 3D model changes that have occurred, along with the illustrations.

No.	Illustration and Description
1	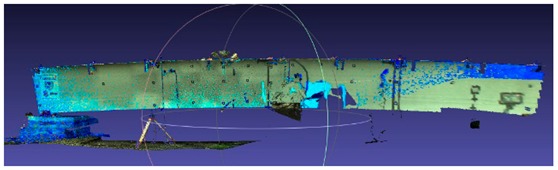
	Both scans were superimposed on one another and formed into two interpenetrating mesh models. Using the compartments of two overlapped schemes, the state of change is visible in the course of a composite bridge span overloading.
2	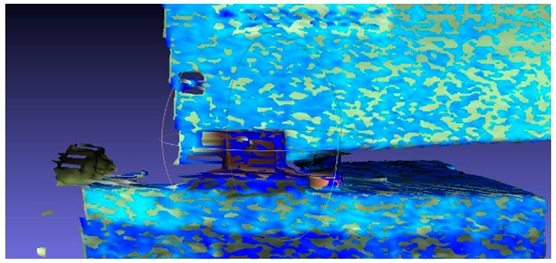
	Bridge span support region, where it can be seen that two mesh models penetrate each other in a uniform manner, indicating that this place shifted after the proof loading process.
3	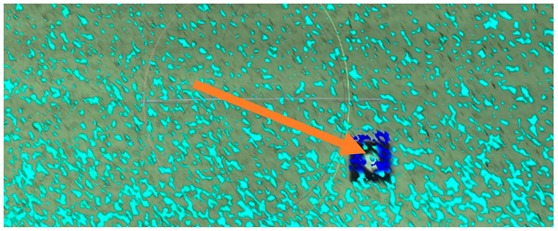
	Bridge span the middle-bottom region, where it is visible that the two mesh models interpenetrate in a homogenous way. This indicates that this place does not shift in the perpendicular direction, but it might be seen, by looking at signal placed to the bridge, that the surface has moved in the vertical direction by a small amount.
4	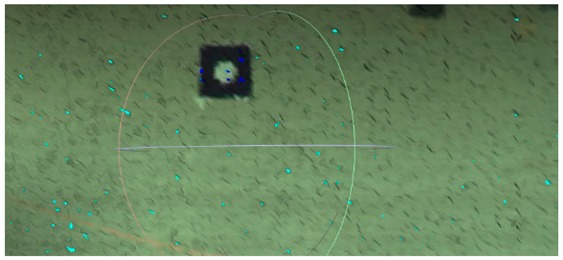
	Bridge span middle-bottom region, in which it is visible that the two mesh models do not interpenetrate, indicating that this place does not shift in the perpendicular direction.
5	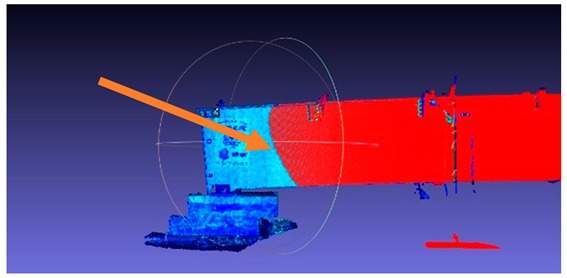
	On the illustration above the mesh model corresponding to the more deformed state is presented in red color. The character of the deformation is visible. Displacement of the side surface has occurred with tilting in the perpendicular direction, determined by the three-dimensional polyline in a parabolic shape. The deformation silhouette may indicate a place where increased stresses start to occur. The elliptical shape of the polyline is puzzling. We can explain it by the increased rigidity of the upper part of the lateral surface caused by the diaphragm lip, perpendicular to the plane of the shell, which closes the top.
6	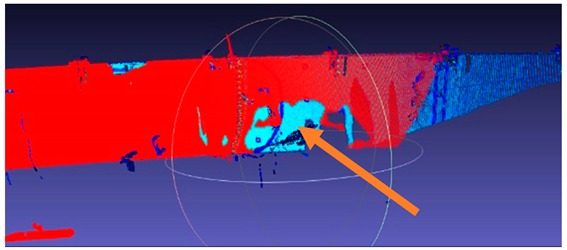
	Obstacles placed between the scanned object and a scanner device cause rifts in the point cloud structure. The breaches, so-called shadows, have been caused by people who passed through in front of the lateral surface of the bridge span during measurement.
7	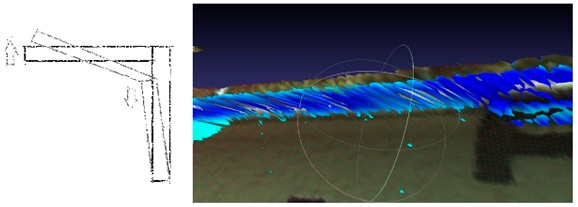
	The diaphragm lip of the bridge is subject to rotation.

**Table 3:** Spheres translation method (STM) example: position change of the sphere SD2 in time (mm).

	Initial	Deformed
Sphere code	Sphere SD2_S1	Sphere SD2_S2
Set points zone	0.01	0.02
X	−1.76	−1.77
Y	1.94	1.95
Z	0.05	0.05

**Table 4:** Proof loading of the composite bridge: load combinations [8].

Load Scheme	Image	Load Scheme	Image
U1: 1 + 2	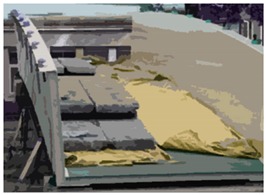	U1: 2 + 3 + 4	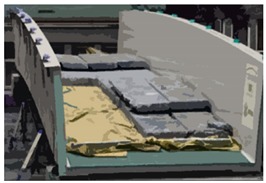
U1: 1 + 2 + 3	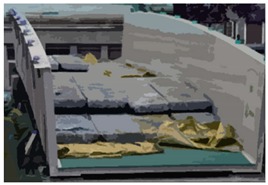	U1: 3 + 4	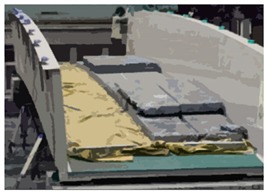
U1: 1 + 2 + 3 + 4	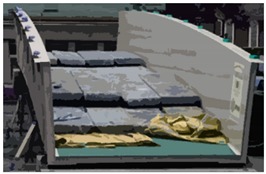	U1: 4	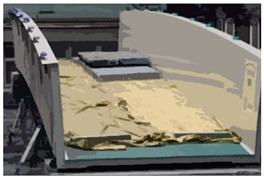

## Appendix A.

We listed acronyms used in the paper in Table A1.

**Table A1:** List of acronyms used in the paper.

No.	Acronym	Description
1	FM	Fast Marching algorithm
2	ICP	Iterative Closest Point
3	KD-Tree	KD-Tree algorithm
4	RANSAC	RANdom SAmple Consensu
5	STM	Spheres Translation Method
6	TIN	Triangulated Irregular Network
7	TLS	Terrestrial Laser Scanning
